# Geotemporospatial and causal inferential epidemiological overview and survey of USA cannabis, cannabidiol and cannabinoid genotoxicity expressed in cancer incidence 2003–2017: part 1 – continuous bivariate analysis

**DOI:** 10.1186/s13690-022-00811-8

**Published:** 2022-03-30

**Authors:** Albert Stuart Reece, Gary Kenneth Hulse

**Affiliations:** 1 Division of Psychiatry, University of Western Australia, Crawley, WA 6009 Australia; 2 School of Medical and Health Sciences, Edith Cowan University, Joondalup, WA 6027 Australia; 3 Brisbane, Australia

**Keywords:** Cannabis, Cannabinoid, Δ9-tetrahydrocannabinol, Cannabigerol, Cannabidiol, Mechanisms, Congenital anomalies, Oncogenesis, Genotoxicity, Epigenotoxicity, Chromosomal toxicity, Multigenerational genotoxicity, Transgenerational teratogenicity, Dose–response relationship, Supra-linear dose response, Sigmoidal dose–response

## Abstract

**Background:**

The genotoxic and cancerogenic impacts of population-wide cannabinoid exposure remains an open but highly salient question. The present report examines these issues from a continuous bivariate perspective with subsequent reports continuing categorical and detailed analyses.

**Methods:**

Age-standardized state census incidence of 28 cancer types (including “All (non-skin) Cancer”) was sourced using SEER*Stat software from Centres for Disease Control and National Cancer Institute across US states 2001–2017. It was joined with drug exposure data from the nationally representative National Survey of Drug Use and Health conducted annually by the Substance Abuse and Mental Health Services Administration 2003–2017, response rate 74.1%. Cannabinoid data was from Federal seizure data. Income and ethnicity data sourced from the US Census Bureau. Data was processed in R.

**Results:**

Nineteen thousand eight hundred seventy-seven age-standardized cancer rates were returned. Based on these rates and state populations this equated to 51,623,922 cancer cases over an aggregated population 2003–2017 of 124,896,418,350. Regression lines were charted for cancer-substance exposures for cigarettes, alcohol use disorder (AUD), cannabis, THC, cannabidiol, cannabichromene, cannabinol and cannabigerol. In this substance series positive trends were found for 14, 9, 6, 9, 12, 6, 9 and 7 cancers; with largest minimum E-Values (mEV) of 1.76 × 10^9^, 4.67 × 10^8^, 2.74 × 10^4^, 4.72, 2.34 × 10^18^, 2.74 × 10^17^, 1.90 × 10^7^, 5.05 × 10^9^; and total sum of exponents of mEV of 34, 32, 13, 0, 103, 58, 25, 31 indicating that cannabidiol followed by cannabichromene are the most strongly implicated in environmental carcinogenesis. Breast cancer was associated with tobacco and all cannabinoids (from mEV = 3.53 × 10^9^); “All Cancer” (non-skin) linked with cannabidiol (mEV = 1.43 × 10^11^); pediatric AML linked with cannabis (mEV = 19.61); testicular cancer linked with THC (mEV = 1.33). Cancers demonstrating elevated mEV in association with THC were: thyroid, liver, pancreas, AML, breast, oropharynx, CML, testis and kidney. Cancers demonstrating elevated mEV in relation to cannabidiol: prostate, bladder, ovary, all cancers, colorectum, Hodgkins, brain, Non-Hodgkins lymphoma, esophagus, breast and stomach.

**Conclusion:**

Data suggest that cannabinoids including THC and cannabidiol are important community carcinogens exceeding the effects of tobacco or alcohol. Testicular, (prostatic) and ovarian tumours indicate mutagenic corruption of the germline in both sexes; pediatric tumourigenesis confirms transgenerational oncogenesis; quantitative criteria implying causality are fulfilled.

**Supplementary Information:**

The online version contains supplementary material available at 10.1186/s13690-022-00811-8.

## Background

Aside from testicular carcinogenesis the relationship of cannabis use to cancer incidence is controversial with both positive [1–19] and negative [4, 20, 21] case–control studies being well described [17, 22–25]. Cannabis use has been associated with cancer of the head and neck [1–3], lung [4–6], larynx [7], prostate [7], testes [8–11], cervix [7], brain [12] and urothelial tracts [13–15]. Some investigators have described evidence of a positive dose–response relationships [1, 3, 4, 6]. Several paediatric cancers have been found to be elevated following prenatal in utero exposure including childhood neuroblastoma [16], rhabdomyosarcoma [17] and leukaemia particularly non-lymphoblastic leukaemia [18] which provide clinical evidence of inheritable mutagenicity and carcinogenicity [26, 27].

The increasing use of cannabis internationally [28] associated with rising cannabinoid concentrations [29–31], increasing intensity of near daily use [32] and the prolonged storage time of cannabinoids in adipose reservoirs and gonads [33–38] in a context where laboratory studies have long indicated that cannabinoid genotoxicity is more significant with higher dose exposures and a pseudo-exponential genotoxic dose–response curve [39–44] imply that there is a pressing need to apply novel and innovative epidemiological methodologies to the investigation of this important issue at the level of population health.

The case of testicular carcinogenesis is at once interesting, important and instructive. Since it is well established that the testicular germ cell niche begins its oncogenic transformation during in utero life [45–47] and the mean age of testicular carcinoma incidence is 34 years [48] this implies a protracted period of subclinical transformation of the germ cell epithelium. Since cannabis exposure in adolescent and young adult life is known to increase the incidence of testicular cancer [8–11, 49, 50] by an average of 2.59-fold (95%C.I. 1.60–4.19) in meta-analysis [50] it follows that cannabis exposure dramatically accelerates testicular carcinogenesis by 2.4-fold from 34 to around 12 years [51]. The molecular pathogenesis of testicular carcinogenic transformation involves key oncogenic steps including whole genome reduplication, loss of arms of dozens of chromosomes, widespread genome demethylation and functional or structural reduplication events on chromosome 12. Since cannabis accelerates this pathway so markedly it follows that it must have diverse major genotoxic effects in the human testicular germ cell niche. Since the testis houses the male germ cell epithelium the possibility remains open that such major genotoxic damage may be passed on to subsequent generations through the male germ line.

Indeed the recent demonstration that cannabis is causally linked with several birth defects including trisomies of chromosomes 21, 18, and 13, deletion 22q11.2 [52] along with paediatric acute lymphoid leukaemia [53] (which also involves damage to chromosome 12) directly implicates cannabis exposure in damage to 528 MB of the human genome representing 17.6% of its 3,000 MB total length [52]. Indeed one recent study has provided epidemiological evidence that cannabis exposure is causally linked to breast, thyroid, pancreatic and liver cancer along with acute myeloid leukaemia in USA [52]. The link with paediatric acute non-lymphoid leukaemia has previously been demonstrated also by earlier researchers [18, 54]. Since acute lymphoid leukaemia is the commonest cancer of childhood it follows that cannabis may also be an important cause and driver of rising rates of paediatric cancer rates and this has also recently been demonstrated [55].

Some widely quoted earlier negative case–control studies suffered major methodological limitations such as the deletion of individuals who accumulated a high lifetime cannabis exposure from the analysis [21] which, given what has been learned since that time, amounts to a virtual amputation of the signal of interest.

Cannabidiol is of particular interest and concern as it is widely promoted in the culture for a myriad of medical complaints as it is not hallucinogenic and is said not to be psychoactive. However it is widely recommended for the relief of anxiety and it is likely that it is acting at the cannabinoid type 1 receptor (CB1R) where it has been shown to bind after high dose exposure [56–60]. Moreover it was shown long ago that many cannabinoids including Δ9-tetrahydrocannabinol, cannabidiol and cannabinol are genotoxic [38] and indeed the genotoxic moiety was demonstrated to be the polycyclic central ring shared by all cannabinoids known as olevitol [36]. Cannabidiol and many other cannabinoids inhibit mitochondrial function directly through the mitochondrial cannabinoid signalling system, by reactive oxygen species generation and through uncoupling protein 2 [61–67]. Mitochondria are a key regulator of epigenetic function via both indirect regulatory pathways and substrate supply [68]. With the major popular focus on cannabidiol relating to alleged lack of psychoactive potency the far-reaching implications of its known genotoxicity and epigenotoxicity have been essentially overlooked.

The broad issue then of the relationship between cannabis and cancer incidence must be regarded as an open question. A formal detailed epidemiological exploration of this large issue is necessarily complex so the matter has been broken into a series of three papers to aid with presentation and to assist reader understanding. The present paper considers cancer and drug exposures as continuous variables. It is followed by a second paper which examines these covariates as categorical variables which allows data dichotomization in various ways and the calculation of key parameters of interest such as attributable fraction in the exposed and population attributable risk and thus allows the derivation of national case numbers affected [69]. Finally two cannabidiol-related cancers are considered in detail as a demonstration of the manner in which advanced statistical methods can be deployed to investigate these questions [70]. Prostate and ovarian cancer were chosen for these examples as their relationship with cannabidiol was amongst the strongest and their role in the reproductive tract may portend transgenerational impacts and these are the subject of the third paper in this series [70]. It is important that all three papers be read collectively to appreciate the depth and the inter-relatedness and therefore the power of the evidence implicating cannabinoid genotoxicity with an important role in epidemiological cancerogenicity.

## Methods

### Data

Rates of age-adjusted cancer rates by state and year and cancer type was taken from the Surveillance, Epidemiology and End Results (SEER) database from the Centres for Disease Control (CDC) Atlanta, Georgia and the National Cancer Institute (NCI) and from the National Program of Cancer Registries (NPCR) and SEER Incidence US Cancer Statistics Public Use Database 2019 submission covering years 2001–2017 using the SEER*Stat software [71]. The focus of this study was 28 of the most common cancers (as listed below). This includes the category all non-skin cancer (called All Cancer in this report). This was joined with drug use cross-tabulation data across USA by state and year from the National Survey of Drug Use and Health (NSDUH) Restricted-Use Data Analysis System (RDAS) of the Substance Use and Mental Health Data Archive (SAMHDA) held by the Substance Use and Mental Health Services Administration (SAMHSA) for 2003–2017 [72]. Thus the overlap period between the cancer and drug exposure datasets was 2003–2017 which therefore became the period of analysis. The variables of interest were last month cigarettes, last year alcohol use disorder (AUD), last month cannabis, last year non-medical use of opioid analgesics (Analgesics) and last year cocaine. Quintiles of substance exposure were calculated for each year numbered from one, the lowest quintile, to five the highest exposure quintile. Data on median household income, ethnicity and population by state and year was sourced directly from the US Census bureau via the tidycensus package [73] in R including linear interpolation for missing years. The ethnicities of interest were Caucasian-American, African-American, Hispanic-American, Asian-American, American Indian / Alaska Native (AIAN) and Native Hawaiian / Pacific Islander (NHPI). Data on cannabinoid concentration across USA was taken from reports published by the US Drug Enforcement Agency (DEA) for the five cannabinoids Δ9-tetrahydrocannabinol (THC), cannabigerol (CBG), cannabichromene (CBC), cannabinol (CBN), and cannabidiol (CBD) [29–31]. It was multiplied by state level cannabis use to provide an estimate of state level exposure. Quintiles of cannabinoid exposure were calculated on the whole period considered in aggregate. These are used particularly in Part 2. Age adjusted case numbers were derived by multiplying the age-adjusted cancer rate in each state and year by the population of that state and dividing it by 10,000.

### Statistical analysis

Data was processed in R-Studio version 1.3.1093 (2009–2020) based upon R version 4.0.3 (2020–10-10). Covariates were log transformed guided by the Shapiro-Wilks test. Data was manipulated using the “dplyr” package in the “tidyverse” [74]. Graphs were drawn in ggplot2 from tidyverse [74, 75] and maps and graphs were drawn in R-Base, ggplot2 and “sf” (simple features) [76]. Some colour palettes employed the viridis and plasma palettes taken from the package “Viridis” [77] and several palettes were originally designed for this project. Bivariate maps were drawn using colorplaner two way colour matrices [78]. All maps and graphs are original and have not been previously published.

### Regression models

Bivariate linear trends were computed with linear regression from R-Base.

### Simultaneous multiple model analysis

This was conducted in the tidyverse package “purrr” [74] using tidy and glance from package “broom” [79] using established nest-map-unnest workflows. In this way a whole long dataset providing data on many cancers could be analyzed in a single analysis run at one time.

### Causal inference

E-values (expected values) quantitate the degree of an association required of some unknown extraneous confounder variable with both the exposure of concern and the outcome of interest to explain away an apparently causal effect. They therefore provide a quantitative estimate of the degree to which the model is formally complete and subject to extraneous explanations from unidentified confounding covariates. They are a foundational pillar for formal quantitative causal inferential methods. E-value estimates above nine are said to be high [80] and a threshold of 1.25 is typically quoted as being required of potentially causal effects [81]. E-values were computed using the R-package “EValue” [82] from regression equations using the parameter estimate, its standard error and the standard model deviation [81, 83, 84].

*P* < 0.05 was considered significant throughout.

### Data availability

Data, including R-code, ipw weights and spatial weights has been made freely available through the Mendeley Data repository online and can be accessed at http://dx.doi.org/10.17632/dt4jbz7vk4.1

### Ethics

Ethical approval for this study was granted from the University of Western Australia Human Research Ethics Committee approval number on 7^th^ January 2020 RA/4/20/7724.

## Results

The cancers upon which we chose to focus our attention were chosen because they were relatively common or because they involved tissues which had been implicated in the literature with cannabinoid activities. For this reason cancers of the male and female reproductive tract were well represented amongst the cancers chosen for study. The list in alphabetical order includes tumours of: acute lymphoid leukaemia (ALL), acute myeloid leukaemia (AML), bladder, brain, breast, cervix, chronic lymphoid leukaemia (CLL), chronic myeloid leukaemia (CML), colorectum, oesophagus, Hodgkins lymphoma, Kaposi sarcoma, kidney, liver, lung, melanoma, multiple myeloma, Non-Hodgkins lymphoma, oropharynx, ovary, pancreas, penis, prostate, stomach, testis, thyroid and vulva and vagina combined. Based on 2017 data the 27 cancers chosen comprehended 1,339,737 of the 1,670,227 cancers reported to state cancer registries in that year or 80.21% of all non-melanoma non-skin cancers reported. In addition total non-skin cancer was also included in this list making 28 cancer types in all.

19,877 age-adjusted cancer rates were retrieved from the SEER*Stat State NPCR database. The total age-adjusted number of cancers reviewed across the 28 cancer types was 51,623,922 and the total aggregated population across the period 2003–2017 was 124,896,418,350.

Other papers in this series consider categorical [69] and detailed analyses [70] respectively.

### Bivariate continuous analysis

**Fig. 1:**
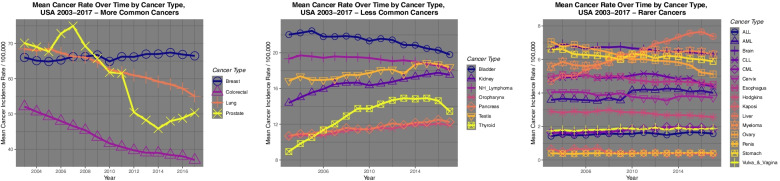
Time trends of **A** common, **B** intermediate frequency and **C** rarer cancers in USA 2003–2017

Figure 1 shows the time trend for the age-adjusted incidence rate for more common cancers (panel A), less common cancers (panel B) and rare cancers (panel C) derived from the CDC SEER*Stat database.

**Fig. 2:**
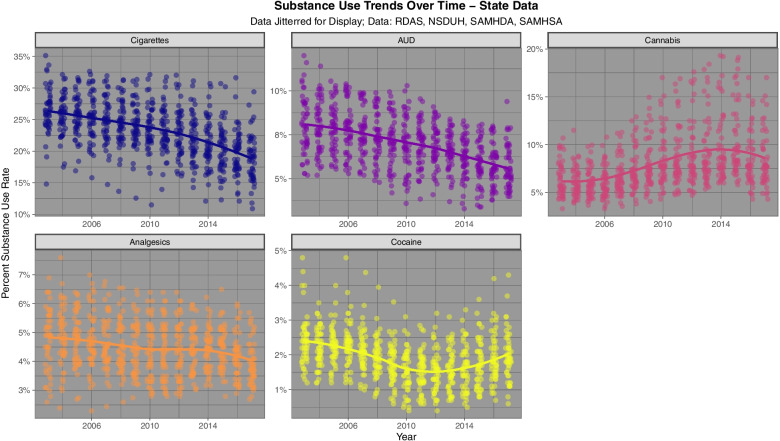
Trends in various substance use rates at state level across USA 2003–2017

The NSDUH survey reports a national response rate of 74.1% [85]. Figure 2 shows the time trend for five substances of interest. One notes that cannabis alone shows a strong upward trend whilst the rate of the other substances is falling or in the case of cocaine, variable and at a low level.

**Fig. 3:**
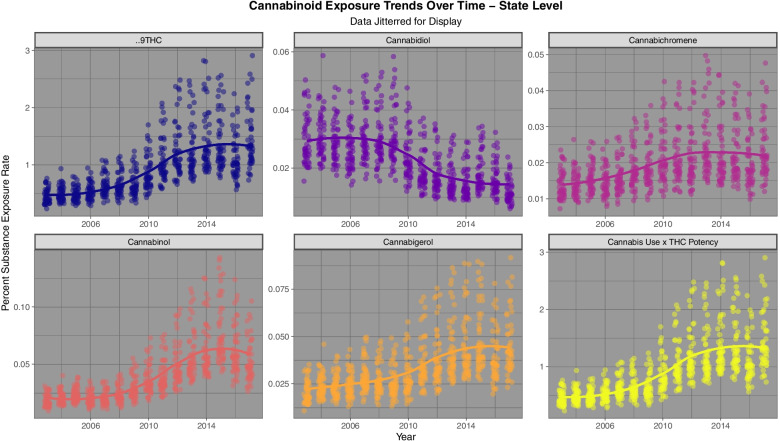
State level cannabinoid exposure estimates across USA 2003 – 2017

Fig. 3 shows the rate for the state based estimates of cannabinoid exposure calculated as described above.

**Fig. 4:**
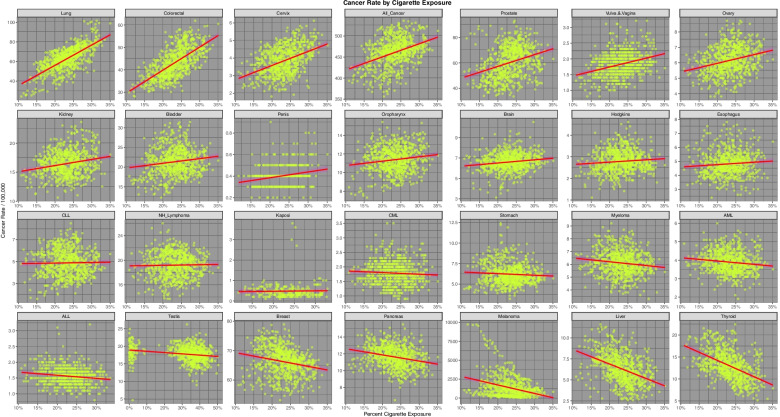
Incidence of 28 cancer types by tobacco exposure across USA

Fig. 4 shows a progression of the incidence rates of 28 cancers of interest, including all cancers, against tobacco exposure. The panels of the graph are ordered by the slope of the cancer:tobacco regression line. The first 9 cancers are seen to be rising in association with increasing tobacco exposure. The fastest rising cancer is lung cancer, which of course is well known. This confirmation of this important finding confirms the technical utility of this technique and indicates that its extension to other substances would also be of interest and of worth. One notes that the top line of the graph also includes cervical cancer, all cancer and vulvovaginal cancer. Bladder, oropharyngeal and esophageal cancer also appear in the second line of the graph which are well established as being tobacco-related tumours.

**Fig. 5:**
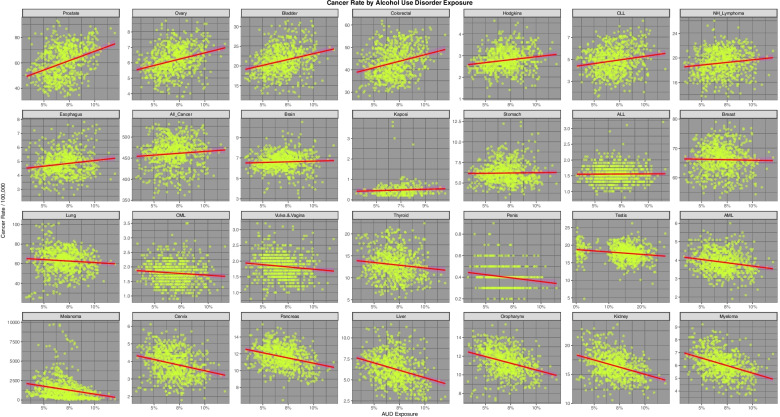
Incidence of 28 cancer types by Alcohol Use Disorder incidence across USA

Fig. 5 presents the relationship of the various tumour incidences to AUD exposure. Esophageal and all cancer are noted to demonstrate positive relationships which are confirmed in the published literature [86].

**Fig. 6:**
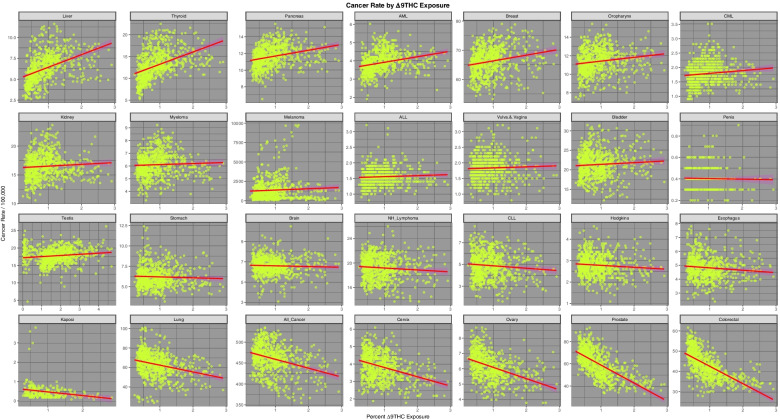
Incidence of 28 cancer types by estimates of Styate level Δ9-Tetrahydrocannabinol exposure across USA

Fig. 6 presents the relationship of the various tumours of interest to THC exposure. 14 tumours are noted to demonstrate a positive relationship as shown in the top two lines.

**Fig. 7:**
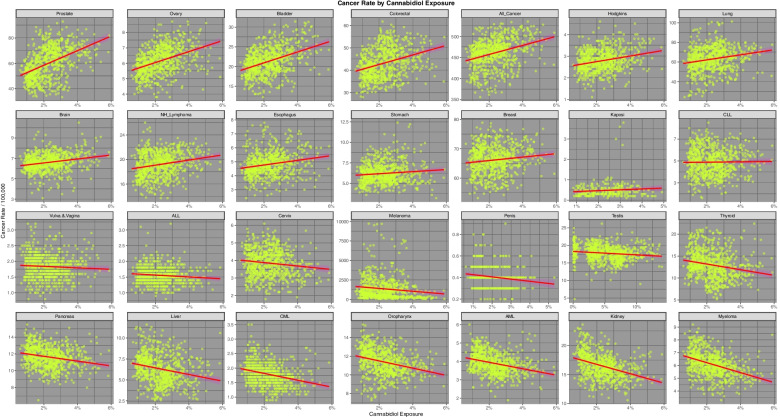
Incidence of 28 cancer types by estimates of Styate level Cannabidiol exposure across USA

Fig. 7 presents the relationship of the various tumours to estimated cannabidiol exposure. 13 cancers are noted to demonstrate a positive relationship, the two most strongly related being prostate and ovarian cancer.

Using the techniques for multiple model simultaneous analysis in R packages purrr and broom it is possible to analyze the slopes of these regression lines for the multiple cancers simultaneously by substance type. The full results of this analysis are shown in Supplementary Table 1 (Excel Sheetname “ST1 Subs Slopes All Canc's”) which lists the slope of the regression line (as the Student’s t statistic) the *P*-value for the significance of the relationship together with various model parameters and their applicable E-Values for all 28 tumours. The table is ordered in terms of descending minimum E-Value. The most significant of these results is shown in Table 1. Table 1 is ordered both my minimum E-value and by substance. One notes that thyroid and liver cancer are the top two associations for cannabis use (*P*-values 2.3510x^−24^ and 4.82 × 10^–20^ and minimum E-Values 2.74 × 10^4^ and 7.96 × 10^3^ respectively) with breast, bladder and pancreatic cancers and AML also featuring significantly.

**Table 1:** Significant Linear Regression Models by Substance

Parameters						Model					E-Values	
**Cancer**	**Substance**	**Estimate**	**Std.Error**	**t-Statistic**	***P*-Value**	**R.Squared**	**Adj.R.Squared**	**Sigma**	**t-Statistic**	***P*-Value**	**E-Value—Point**	**E-Value—Lower**
All_Cancer	Cigarettes	311.2758	26.2010	11.8803	6.10E-30	0.1587	0.1576	30.2276	141.1415	6.10E-30	2.35E + 04	5.02E + 03
All_Cancer	AUD	174.3105	77.5060	2.2490	0.0248	0.0067	0.0054	32.8455	5.0580	0.0248	249.76	3.16
Cervix	Analgesics	14.2232	2.9928	4.7525	2.41E-06	0.0293	0.0280	0.7557	22.5865	2.41E-06	5.49E + 07	4.76E + 04
Ovary	Analgesics	13.0281	3.4213	3.8079	1.52E-04	0.0190	0.0177	0.8639	14.5002	1.52E-04	1.82E + 06	1.58E + 03
Lung	Analgesics	156.2452	47.8675	3.2641	0.0011	0.0140	0.0127	12.0870	10.6545	0.0011	2.57E + 05	222.51
Prostate	AUD	294.0806	26.9078	10.9292	6.73E-26	0.1377	0.1365	11.4030	119.4472	6.73E-26	3.11E + 10	4.67E + 08
Ovary	AUD	16.4306	1.9686	8.3461	3.40E-16	0.0852	0.0840	0.8343	69.6578	3.40E-16	1.21E + 08	1.82E + 06
Colorectal	AUD	116.6256	14.8666	7.8448	1.50E-14	0.0760	0.0748	6.3002	61.5409	1.50E-14	4.14E + 07	6.21E + 05
Bladder	AUD	59.4761	7.6578	7.7668	2.65E-14	0.0746	0.0734	3.2452	60.3227	2.65E-14	3.50E + 07	5.25E + 05
CLL	AUD	13.3118	2.7643	4.8156	1.78E-06	0.0301	0.0288	1.1714	23.1903	1.78E-06	6.19E + 04	928.09
Hodgkins	AUD	5.4767	1.1398	4.8051	1.87E-06	0.0299	0.0286	0.4830	23.0892	1.87E-06	6.06E + 04	907.37
Esophagus	AUD	8.1151	2.0465	3.9653	8.06E-05	0.0214	0.0201	0.8377	15.7240	8.06E-05	1.35E + 04	173.64
NH_Lymphoma	AUD	17.1252	4.6200	3.7067	2.25E-04	0.0180	0.0167	1.9579	13.7398	2.25E-04	5.73E + 03	85.34
Thyroid	Cannabis	38.1264	3.6137	10.5504	2.35E-24	0.1295	0.1284	2.9671	111.3120	2.35E-24	2.40E + 05	2.74E + 04
Liver	Cannabis	18.7374	1.9860	9.4349	4.82E-20	0.1064	0.1052	1.6306	89.0176	4.82E-20	6.96E + 04	7.96E + 03
Breast	Cannabis	38.4836	5.4934	7.0054	5.50E-12	0.0616	0.0603	4.5104	49.0761	5.50E-12	4.71E + 03	538.42
Bladder	Cannabis	25.5306	4.0013	6.3805	3.09E-10	0.0516	0.0503	3.2853	40.7110	3.09E-10	2.36E + 03	269.10
Pancreas	Cannabis	6.9457	1.5165	4.5800	5.44E-06	0.0273	0.0260	1.2451	20.9766	5.44E-06	319.83	36.14
AML	Cannabis	2.7773	0.6876	4.0389	5.92E-05	0.0213	0.0200	0.5646	16.3128	5.92E-05	175.35	19.61
Lung	Cigarettes	206.6791	7.3636	28.0678	5.75E-119	0.5130	0.5123	8.4952	787.7991	5.75E-119	8.24E + 09	1.76E + 09
Colorectal	Cigarettes	102.7662	4.2610	24.1177	1.60E-95	0.4375	0.4367	4.9159	581.6646	1.60E-95	3.65E + 08	7.81E + 07
Cervix	Cigarettes	8.1118	0.5950	13.6326	5.88E-38	0.1990	0.1979	0.6865	185.8480	5.88E-38	9.36E + 04	2.00E + 04
Prostate	Cigarettes	92.4997	10.0923	9.1653	4.68E-19	0.1010	0.0998	11.6433	84.0036	4.68E-19	2.76E + 03	589.25
Vulva.&.Vagina	Cigarettes	2.8291	0.3193	8.8614	6.79E-18	0.1031	0.1018	0.3601	78.5253	6.79E-18	2.54E + 03	524.66
Ovary	Cigarettes	5.6544	0.7272	7.7751	2.50E-14	0.0748	0.0735	0.8390	60.4529	2.50E-14	921.01	196.48
Kidney	Cigarettes	10.5734	1.7983	5.8796	6.19E-09	0.0442	0.0429	2.0747	34.5703	6.19E-09	206.12	43.66
Penis	Cigarettes	0.5348	0.1174	4.5549	6.90E-06	0.0476	0.0453	0.1023	20.7467	6.90E-06	231.92	29.65
Brain	Cigarettes	3.1188	0.7234	4.3113	1.84E-05	0.0242	0.0229	0.8346	18.5875	1.84E-05	59.47	12.30
Bladder	Cigarettes	11.8284	2.8920	4.0901	4.78E-05	0.0219	0.0206	3.3364	16.7287	4.78E-05	49.86	10.24
Oropharynx	Cigarettes	4.6826	1.1454	4.0882	4.82E-05	0.0219	0.0205	1.3214	16.7133	4.82E-05	49.79	10.22
Hodgkins	Cigarettes	1.0813	0.4232	2.5549	0.0108	0.0087	0.0073	0.4883	6.5277	0.0108	14.49	2.59
Esophagus	Cigarettes	1.6896	0.7647	2.2095	0.0275	0.0068	0.0054	0.8440	4.8819	0.0275	11.84	1.77
Stomach	Cocaine	45.7579	6.4211	7.1262	2.43E-12	0.0636	0.0623	1.2139	50.7829	2.43E-12	1.58E + 15	1.29E + 11
Ovary	Cocaine	27.1820	4.5057	6.0328	2.53E-09	0.0464	0.0451	0.8518	36.3948	2.53E-09	8.18E + 12	6.66E + 08
Breast	Cocaine	126.7521	24.1889	5.2401	2.09E-07	0.0354	0.0341	4.5728	27.4586	2.09E-07	1.80E + 11	1.47E + 07
Kaposi	Cocaine	14.8095	4.4075	3.3601	8.98E-04	0.0422	0.0385	0.4019	11.2900	8.98E-04	7.32E + 14	2.43E + 06
Bladder	Cocaine	84.8740	17.5732	4.8298	1.66E-06	0.0302	0.0289	3.3221	23.3265	1.66E-06	2.50E + 10	2.04E + 06
Prostate	Cocaine	225.7327	64.4301	3.5035	4.86E-04	0.0161	0.0148	12.1802	12.2747	4.86E-04	4.22E + 07	3.44E + 03
Melanoma	Cocaine	21,958.5719	7636.0557	2.8756	0.0041	0.0109	0.0096	1443.5593	8.2693	0.0041	2.05E + 06	166.80
Hodgkins	Cocaine	5.5031	2.5863	2.1278	0.0337	0.0060	0.0047	0.4889	4.5275	0.0337	5.61E + 04	4.00
Colorectal	Cocaine	65.7878	34.5865	1.9021	0.0575	0.0048	0.0035	6.5384	3.6181	0.0575	1.89E + 04	1.00

Supplementary Table 2 (Excel Sheetname “ST2 Cannbd Slopes All Cancs”) performs a similar function for cannabinoid exposure as Supplementary Table 1. Again the most significant results from this table are extracted as Table 2. 13 of the 44 cancers listed in this Table demonstrate a significant relationship to cannabidiol exposure. The counts for the other cannabinoids are THC = 9, Cannabinol = 9, cannabigerol = 7 and cannabichromene = 6. The most tightly related cancers to cannabidiol exposure are prostate, bladder, ovary and all cancers which have *P*-values ranging from 6.87 × 10^–20^ to 2.23 × 10^–41^ and minimum E-Values ranging from 1.43 × 1011 to 2.34 × 10^18^.

**Table 2:** Significant Linear Regression Models by Cannabinoid

Parameters							Model				E-Values	
**Cancer**	**Substance**	**Term**	**Estimate**	**Std.Error**	**t-Statistic**	***P*-Value**	**Adj.R.Squared**	**Sigma**	**t-Statistic**	***P*-Value**	**E-Value—Point**	**E-Value—Lower**
Prostate	Cannabidiol	CannbdRt	575.7886	40.1598	14.3374	2.28E-41	0.2145	10.8759	205.5620	2.28E-41	1.67E + 21	2.34E + 18
Thyroid	Cannabichromene	CannbdRt	156.7231	14.5353	10.7822	2.70E-25	0.1334	2.9586	116.2561	2.70E-25	1.72E + 21	2.74E + 17
Bladder	Cannabidiol	CannbdRt	138.8861	11.3748	12.2100	2.15E-31	0.1651	3.0805	149.0832	2.15E-31	1.32E + 18	1.84E + 15
Ovary	Cannabidiol	CannbdRt	35.7024	2.9444	12.1254	5.09E-31	0.1632	0.7974	147.0245	5.09E-31	9.91E + 17	1.38E + 15
Liver	Cannabichromene	CannbdRt	76.1608	8.0037	9.5157	2.42E-20	0.1068	1.6291	90.5482	2.42E-20	5.99E + 18	9.53E + 14
All_Cancer	Cannabidiol	CannbdRt	1081.1070	115.0938	9.3933	6.87E-20	0.1043	31.1692	88.2334	6.87E-20	1.02E + 14	1.43E + 11
Colorectal	Cannabidiol	CannbdRt	213.8584	22.9037	9.3373	1.10E-19	0.1032	6.2027	87.1847	1.10E-19	8.46E + 13	1.18E + 11
Thyroid	Cannabigerol	CannbdRt	83.9005	7.2622	11.5530	1.59E-28	0.1503	2.9295	133.4725	1.59E-28	4.17E + 11	5.05E + 09
Liver	Cannabigerol	CannbdRt	45.1026	3.9451	11.4325	5.22E-28	0.1476	1.5914	130.7011	5.22E-28	3.17E + 11	3.85E + 09
Breast	Cannabichromene	CannbdRt	149.2417	22.2143	6.7183	3.64E-11	0.0556	4.5216	45.1350	3.64E-11	2.22E + 13	3.53E + 09
Hodgkins	Cannabidiol	CannbdRt	13.1216	1.7461	7.5146	1.63E-13	0.0690	0.4729	56.4697	1.63E-13	1.85E + 11	2.59E + 08
Bladder	Cannabichromene	CannbdRt	97.3604	16.1873	6.0146	2.82E-09	0.0449	3.2948	36.1757	2.82E-09	9.54E + 11	1.52E + 08
Liver	Cannabinol	CannbdRt	31.8217	2.3327	13.6415	5.33E-38	0.1981	1.5435	186.0893	5.33E-38	2.81E + 08	1.91E + 07
Thyroid	Cannabinol	CannbdRt	58.0845	4.3115	13.4721	3.40E-37	0.1942	2.8528	181.4988	3.40E-37	2.23E + 08	1.51E + 07
Brain	Cannabidiol	CannbdRt	19.4078	3.0380	6.3884	2.94E-10	0.0505	0.8227	40.8119	2.94E-10	4.21E + 09	5.88E + 06
Lung	Cannabidiol	CannbdRt	258.5289	43.9435	5.8832	6.07E-09	0.0429	11.9006	34.6121	6.07E-09	7.70E + 08	1.08E + 06
NH_Lymphoma	Cannabidiol	CannbdRt	41.7851	7.1339	5.8573	7.05E-09	0.0426	1.9320	34.3079	7.05E-09	7.06E + 08	9.87E + 05
Pancreas	Cannabichromene	CannbdRt	28.7938	6.1125	4.7106	2.94E-06	0.0275	1.2442	22.1900	2.94E-06	2.80E + 09	4.46E + 05
Esophagus	Cannabidiol	CannbdRt	16.7282	3.1067	5.3846	9.84E-08	0.0375	0.8303	28.9943	9.84E-08	1.84E + 08	2.35E + 05
AML	Cannabichromene	CannbdRt	12.1345	2.7686	4.3830	1.34E-05	0.0237	0.5635	19.2103	1.34E-05	6.47E + 08	1.03E + 05
Breast	Cannabigerol	CannbdRt	74.3296	11.2175	6.6262	6.59E-11	0.0542	4.5251	43.9067	6.59E-11	6.21E + 06	7.52E + 04
Pancreas	Cannabigerol	CannbdRt	19.4504	3.0478	6.3818	3.07E-10	0.0504	1.2294	40.7277	3.07E-10	3.58E + 06	4.34E + 04
Pancreas	Cannabinol	CannbdRt	14.6579	1.8311	8.0047	4.57E-15	0.0777	1.2116	64.0760	4.57E-15	1.21E + 05	8.20E + 03
AML	Cannabigerol	CannbdRt	7.5386	1.3877	5.4326	7.52E-08	0.0367	0.5598	29.5133	7.52E-08	4.20E + 05	5.09E + 03
AML	Cannabinol	CannbdRt	6.2884	0.8313	7.5646	1.14E-13	0.0698	0.5501	57.2228	1.14E-13	6.59E + 04	4.48E + 03
Breast	Cannabinol	CannbdRt	39.0905	6.8898	5.6737	2.00E-08	0.0400	4.5589	32.1904	2.00E-08	4.89E + 03	331.75
Breast	Cannabidiol	CannbdRt	58.3586	17.0595	3.4209	6.58E-04	0.0141	4.6200	11.7025	6.58E-04	1.96E + 05	274.11
Bladder	Cannabigerol	CannbdRt	31.9672	8.2808	3.8604	1.23E-04	0.0182	3.3404	14.9026	1.23E-04	1.21E + 04	146.30
Oropharynx	Cannabinol	CannbdRt	9.3388	1.9902	4.6924	3.21E-06	0.0273	1.3169	22.0189	3.21E-06	1.27E + 03	85.68
Stomach	Cannabidiol	CannbdRt	12.7562	4.6084	2.7680	0.0058	0.0088	1.2480	7.6621	0.0058	2.19E + 04	30.11
CML	Cannabinol	CannbdRt	2.3785	0.6433	3.6974	2.36E-04	0.0184	0.3850	13.6708	2.36E-04	552.10	27.73
Testis	Cannabinol	CannbdRt	8.0518	1.8461	4.3615	1.47E-05	0.0235	2.1897	19.0228	1.47E-05	56.28	12.14
Oropharynx	Cannabigerol	CannbdRt	8.5809	3.2973	2.6024	0.0094	0.0076	1.3301	6.7725	0.0094	708.47	8.06
Kidney	Cannabinol	CannbdRt	8.6788	3.1913	2.7195	0.0067	0.0085	2.1117	7.3957	0.0067	83.70	5.16
Thyroid	THC	CannbdRt	2.8220	0.2096	13.4665	3.62E-37	0.1941	2.8531	181.3454	3.62E-37	4.35	3.74
Liver	THC	CannbdRt	1.5118	0.1140	13.2611	3.36E-36	0.1893	1.5521	175.8566	3.36E-36	4.29	3.68
Pancreas	THC	CannbdRt	0.6960	0.0892	7.8048	2.01E-14	0.0741	1.2140	60.9153	2.01E-14	2.76	2.32
AML	THC	CannbdRt	0.3032	0.0404	7.5007	1.80E-13	0.0687	0.5504	56.2607	1.80E-13	2.69	2.25
Breast	THC	CannbdRt	1.9686	0.3343	5.8882	5.89E-09	0.0430	4.5517	34.6715	5.89E-09	2.33	1.93
Oropharynx	THC	CannbdRt	0.4108	0.0970	4.2358	2.56E-05	0.0221	1.3204	17.9420	2.56E-05	1.99	1.60
CML	THC	CannbdRt	0.0974	0.0317	3.0756	0.0022	0.0124	0.3862	9.4592	0.0022	1.83	1.39
Testis	THC	CannbdRt	0.3272	0.0880	3.7205	2.14E-04	0.0169	2.1972	13.8421	2.14E-04	1.55	1.33
Kidney	THC	CannbdRt	0.3065	0.1555	1.9716	0.0490	0.0038	2.1166	3.8873	0.0490	1.54	1.03
Kaposi	Cannabidiol	CannbdRt	4.2681	2.8705	1.4869	0.1383	0.0047	0.4089	2.2108	0.1383	2.67E + 04	1.00

Table 3 presents the slopes of the regression lines as the Student’s-t value for each of the substances for the cancers listed in descending order of cannabis slope (as the t-statistic).

**Table 3:** Linear Regression Line Slopes as Student’s t Value by SubstanceOrdered by Slope of Cannabis Regression Line

No. 1	**Cancer**	**Cigarettes Slope**	**AUD Slope**	**Cannabis Slope**	**Analgesics Slope**	**Cocaine Slope**
	Thyroid	-7.9274	-3.3481	11.8389	-15.5097	-0.8113
2	Liver	1.7715	-9.1320	11.2877	-12.7769	1.7084
3	Breast	-5.2344	-0.5894	8.1969	-5.9799	5.2401
4	Bladder	-3.1171	7.7668	6.8791	4.0901	4.8298
5	Pancreas	-4.6223	-8.7651	5.8977	-6.9332	-3.1707
6	AML	-6.5556	-5.4368	5.2288	-3.6910	-7.7492
7	Oropharynx	-3.0980	-9.7255	1.5995	4.0882	-9.5481
8	Melanoma	-0.8915	-6.1055	1.5103	-9.4405	2.8756
9	ALL	-1.5791	0.2444	1.2943	-3.8405	-0.4499
10	CML	-7.4508	-2.4145	1.1710	-1.6592	-7.0446
11	Stomach	0.0693	0.5000	0.9082	-1.8030	7.1262
12	Brain	-0.8121	1.3398	0.8300	4.3113	-0.4800
13	Hodgkins	-6.7766	4.8051	0.8200	2.5549	2.1278
14	Vulva.&.Vagina	-4.6924	-3.1052	0.7639	8.8614	-5.7031
15	NH_Lymphoma	-5.8607	3.7067	0.5957	0.5524	0.3994
16	Esophagus	1.6594	3.9653	-0.8874	2.2095	-0.3545
17	Kidney	-3.5063	-10.5835	-1.5084	5.8796	-9.0454
18	Penis	-0.8998	-3.5026	-1.7672	4.5549	-3.8333
19	Testis	-5.5040	-4.9294	-1.9274	-5.8386	-0.5264
20	Myeloma	-7.3545	-11.4709	-2.1695	-3.6686	-7.4129
21	CLL	-7.8892	4.8156	-2.2216	0.4953	-5.7156
22	Kaposi	0.9951	0.9317	-3.1931	0.3724	3.3601
23	All_Cancer	-3.3716	2.2490	-3.8878	11.8803	1.6573
24	Lung	3.2641	-2.0936	-4.4455	28.0678	0.3049
25	Ovary	3.8079	8.3461	-6.5747	7.7751	6.0328
26	Cervix	4.7525	-7.3111	-10.4359	13.6326	-1.8426
27	Prostate	1.0881	10.9292	-13.8019	9.1653	3.5035
28	Colorectal	0.6247	7.8448	-16.1417	24.1177	1.9021

Table 4 performs a similar function for the cannabinoids listed in descending order of the cannabidiol slope (as the t-statistic).

**Table 4:** Linear Regression Line Slopes by Cannabinoid Ordered by Slope of Cannabidiol Regression Line

No	Cancer	THC Slope	Cannabidiol Slope	Cannabichromene Slope	Cannabinol Slope	Cannabigerol Slope
1	Prostate	-25.4999	15.0011	-13.9506	-30.0790	-19.2506
2	Ovary	-14.0584	13.1851	-7.3292	-14.4851	-10.2498
3	Bladder	1.7824	11.7295	6.4769	1.1324	4.2897
4	Colorectal	-26.7535	10.2176	-17.3209	-26.1712	-20.6437
5	All_Cancer	-8.4752	9.2868	-4.2320	-9.1614	-6.3380
6	Hodgkins	-2.4889	7.1408	0.6127	-3.0804	-1.1613
7	Lung	-7.9645	6.5177	-4.7560	-8.0742	-6.3507
8	Brain	-1.7981	5.7887	0.6371	-2.0640	-0.5697
9	NH_Lymphoma	-2.1409	5.7874	0.3796	-2.3523	-0.9701
10	Esophagus	-3.5846	5.5661	-1.1051	-3.6621	-2.2324
11	Stomach	-0.9189	3.6282	0.6205	-1.0002	-0.0239
12	Breast	6.9024	2.9740	7.8292	6.6043	7.8098
13	Kaposi	-3.8468	1.8329	-3.3135	-3.8992	-3.3551
14	CLL	-2.2840	-0.1408	-2.2461	-2.2960	-2.6415
15	Vulva.&.Vagina	1.9115	-2.2486	0.7538	1.7822	1.0338
16	ALL	2.7533	-2.6301	1.1321	2.8397	1.9626
17	Cervix	-9.4408	-2.7163	-10.6708	-8.5617	-10.1059
18	Melanoma	2.9323	-2.7495	1.6322	3.0804	2.3487
19	Penis	0.1269	-3.6579	-1.9384	0.1510	-0.8129
20	Testis	-0.1463	-4.2166	-2.0019	0.4269	-1.3235
21	Thyroid	16.5506	-6.2049	12.2596	16.4519	13.4125
22	Pancreas	10.0878	-7.1535	6.0706	10.3462	8.1530
23	Liver	16.7546	-7.4077	11.4863	17.1885	14.0257
24	CML	4.9482	-8.6931	1.2939	5.6310	2.8365
25	Oropharynx	5.7731	-8.9380	1.8175	6.2778	3.5506
26	AML	9.9660	-9.5158	5.5823	10.4116	7.0927
27	Kidney	4.2116	-12.5177	-0.9390	4.6814	1.4827
28	Myeloma	3.2281	-12.8529	-1.9390	4.1031	0.1689

As noted above Tables 1, 2, 3 and 4 present data for all cancers and all rates. Table 5 takes the logs of the cannabis exposure rate and the cannabinoid exposure rates (as indicated by the Shapiro-Wilks test) and regresses them against the cancer rates for each tumour (using the broom-purrr workflow sequence on the dataset in long format). The table concentrates on those tumours with positive and significant regression line slopes. The results are at once intriguing and fascinating. Only four tumours namely ALL, CML, myeloma and testicular cancer, do not appear in this table which is quite remarkable in itself. If one considers this Table in the light of Figs. 1, 2 and 3 one notes that those cancers with falling incidence correlate significantly with those substances whose use is falling. For this reason it is very obvious from the Table that cigarettes, AUD and cannabidiol are grouped together in one cluster whilst all the other cannabinoids, whose exposure is rising, group together in another cluster. The tumours which correlate most tightly with cannabidiol exposure are prostate, ovary, bladder, colorectal and total cancers. Cannabidiol therefore is highly associated with the commonest tumours namely all non-skin cancers, breast, lung and prostate cancer. Interestingly breast cancer correlates with cocaine, cannabis and all the cannabinoids. The substance most associated with cancer types in this table is tobacco (14 tumour types) followed by cannabidiol (12 tumour types) followed by AUD (9 tumour types).

**Table 5:** Summary of Significant Regression Line Positive Slopes by Cancer, Substance and *P*-Value Calculations Utilize Logarithm of Rates of Cannabis and Cannabinoid Exposure

No	Cancer	Cigarettes	AUD	Analgesics	Cocaine	Cannabis	THC	Cannabidiol	Cannabichromene	Cannabinol	Cannabigerol
1	Prostate	4.68E-19	6.73E-26		4.86E-04			1.15E-44			
2	Ovary	2.50E-14	3.40E-16	1.52E-04	2.53E-09			7.61E-36			
3	Bladder	4.78E-05	2.65E-14		1.66E-06	1.27E-11		2.77E-29	1.70E-10		2.02E-05
4	Colorectal	1.60E-95	1.50E-14					4.96E-23			
5	All_Cancer	6.10E-30	2.48E-02					1.69E-19			
6	Hodgkins	1.08E-02	1.87E-06		3.37E-02			2.20E-12			
7	Lung	5.75E-119		1.15E-03				1.31E-10			
8	Brain	1.84E-05						1.04E-08			
9	NH_Lymphoma		2.25E-04					1.05E-08			
10	Esophagus	2.75E-02	8.06E-05					3.68E-08			
11	Stomach				2.43E-12			3.05E-04			
12	Breast				2.09E-07	1.07E-15	1.09E-11	3.03E-03	1.68E-14	7.57E-11	1.94E-14
13	Liver					2.15E-27	9.68E-54		3.08E-28	4.67E-56	7.57E-40
14	Thyroid					9.25E-30	1.16E-52		1.29E-31	3.86E-52	6.51E-37
15	Pancreas					5.58E-09	1.60E-22		2.03E-09	1.54E-23	1.49E-15
16	AML					2.22E-07	4.73E-22		3.32E-08	8.47E-24	3.05E-12
17	Oropharynx	4.82E-05					1.14E-08			5.82E-10	4.08E-04
18	Kidney	6.19E-09					2.84E-05			3.38E-06	
19	Melanoma				4.15E-03		3.47E-03			2.14E-03	1.91E-02
20	Cervix	5.88E-38		2.41E-06							
21	Vulva.&.Vagina	6.79E-18									
22	Penis	6.90E-06									
23	Kaposi				8.98E-04						
24	CLL		1.78E-06								
	**Cancer Numbers**	14	9	3	8	6	8	12	6	8	8

Table 6 extracts the results from Supplementary Table 1 for cigarette exposure. Cancers are again listed in descending order of the minimum E-Value. One notes that the list is headed by lung, cervical, colorectal, All cancer and vulvovaginal cancers which seems correct. Fourteen cancers are noted to be significantly related and all have minimum E-Values > 1.70.

**Table 6:** Summary of Tobacco Regression Line Slopes by Cancer and E-Value

Parameters					Model					E-Values	
**Cancer**	**Estimate**	**Std.Error**	**t-Statistic**	***P*-Value**	**R.Squared**	**Adj.R.Squared**	**S.D**	**t-Statistic**	***P*-Value**	**E-Value—Point**	**E-Value—Lower**
Lung	206.6791	7.3636	28.0678	5.75E-119	0.5130	0.5123	8.4952	787.7991	0.0000	8.24E + 09	1.76E + 09
Colorectal	102.7662	4.2610	24.1177	1.60E-95	0.4375	0.4367	4.9159	581.6646	0.0000	3.65E + 08	7.81E + 07
Cervix	8.1118	0.5950	13.6326	5.88E-38	0.1990	0.1979	0.6865	185.8480	0.0000	9.36E + 04	2.00E + 04
All_Cancer	311.2758	26.2010	11.8803	6.10E-30	0.1587	0.1576	30.2276	141.1415	0.0000	2.35E + 04	5.02E + 03
Prostate	92.4997	10.0923	9.1653	4.68E-19	0.1010	0.0998	11.6433	84.0036	0.0000	2.76E + 03	589.2479
Vulva.&.Vagina	2.8291	0.3193	8.8614	6.79E-18	0.1031	0.1018	0.3601	78.5253	0.0000	2.54E + 03	524.6626
Ovary	5.6544	0.7272	7.7751	2.50E-14	0.0748	0.0735	0.8390	60.4529	0.0000	921.0142	196.4846
Kidney	10.5734	1.7983	5.8796	6.19E-09	0.0442	0.0429	2.0747	34.5703	0.0000	206.1206	43.6624
Penis	0.5348	0.1174	4.5549	6.90E-06	0.0476	0.0453	0.1023	20.7467	0.0000	231.9196	29.6474
Brain	3.1188	0.7234	4.3113	1.84E-05	0.0242	0.0229	0.8346	18.5875	0.0000	59.4650	12.2980
Bladder	11.8284	2.8920	4.0901	4.78E-05	0.0219	0.0206	3.3364	16.7287	0.0000	49.8607	10.2407
Oropharynx	4.6826	1.1454	4.0882	4.82E-05	0.0219	0.0205	1.3214	16.7133	0.0000	49.7858	10.2246
Hodgkins	1.0813	0.4232	2.5549	0.0108	0.0087	0.0073	0.4883	6.5277	0.0108	14.4877	2.5879
Esophagus	1.6896	0.7647	2.2095	0.0275	0.0068	0.0054	0.8440	4.8819	0.0275	11.8432	1.7676
NH_Lymphoma	0.9458	1.7122	0.5524	0.5808	0.0004	-0.0009	1.9754	0.3051	0.5808	2.4649	1.00
CLL	0.5105	1.0309	0.4953	0.6206	0.0003	-0.0010	1.1893	0.2453	0.6206	2.3184	1.00
Kaposi	0.2054	0.5515	0.3724	0.7099	0.0005	-0.0034	0.4105	0.1387	0.7099	2.5299	1.00
CML	-0.5711	0.3442	-1.6592	0.0975	0.0041	0.0026	0.3881	2.7530	0.0975	7.0927	NA
Stomach	-1.9561	1.0849	-1.8030	0.0718	0.0043	0.0030	1.2517	3.2506	0.0718	7.7574	NA
Myeloma	-2.9596	0.8067	-3.6686	0.0003	0.0177	0.0164	0.9307	13.4583	2.61E-04	35.6121	NA
AML	-1.8095	0.4902	-3.6910	0.0002	0.0179	0.0166	0.5656	13.6235	2.40E-04	36.2575	NA
ALL	-0.9906	0.2579	-3.8405	0.0001	0.0233	0.0217	0.2762	14.7494	1.35E-04	51.7645	NA
Testis	-3.6302	0.6218	-5.8386	7.84E-09	0.0436	0.0423	2.1686	34.0895	7.84E-09	8.6444	NA
Breast	-23.5766	3.9426	-5.9799	3.46E-09	0.0456	0.0443	4.5485	35.7597	3.46E-09	223.1309	NA
Pancreas	-7.3544	1.0607	-6.9332	8.90E-12	0.0604	0.0591	1.2238	48.0697	8.90E-12	473.8450	NA
Melanoma	########	1189.3018	-9.4405	4.60E-20	0.1065	0.1053	1372.0760	89.1232	4.60E-20	3.43E + 03	NA
Liver	-17.3073	1.3546	-12.7769	5.90E-34	0.1791	0.1781	1.5628	163.2484	5.90E-34	4.76E + 04	NA
Thyroid	-37.1893	2.3978	-15.5097	3.02E-47	0.2433	0.2423	2.7663	240.5498	3.02E-47	4.11E + 05	NA

Table 7 performs a similar function for last year AUD exposure. Nine cancers are significantly related on this Table and also demonstrate elevated minimum E-Values.

**Table 7:** Summary of Alcohol Use Disorder Regression Line Slopes by Cancer and E-Value

Parameters					Model					E-Values	
**Cancer**	**Estimate**	**Std.Error**	**t-Statistic**	***P*-Value**	**R.Squared**	**Adj.R.Squared**	**S.D**	**t-Statistic**	***P*-Value**	**E-Value—Point**	**E-Value—Lower**
Prostate	294.0806	26.9078	10.9292	6.73E-26	0.1377	0.1365	11.4030	119.4472	6.73E-26	3.11E + 10	4.67E + 08
Ovary	16.4306	1.9686	8.3461	3.40E-16	0.0852	0.0840	0.8343	69.6578	3.40E-16	1.21E + 08	1.82E + 06
Colorectal	116.6256	14.8666	7.8448	1.50E-14	0.0760	0.0748	6.3002	61.5409	1.50E-14	4.14E + 07	6.21E + 05
Bladder	59.4761	7.6578	7.7668	2.65E-14	0.0746	0.0734	3.2452	60.3227	2.65E-14	3.50E + 07	5.25E + 05
CLL	13.3118	2.7643	4.8156	1.78E-06	0.0301	0.0288	1.1714	23.1903	1.78E-06	6.19E + 04	9.28E + 02
Hodgkins	5.4767	1.1398	4.8051	1.87E-06	0.0299	0.0286	0.4830	23.0892	1.87E-06	6.06E + 04	907.3675
Esophagus	8.1151	2.0465	3.9653	8.06E-05	0.0214	0.0201	0.8377	15.7240	8.06E-05	1.35E + 04	173.6406
NH_Lymphoma	17.1252	4.6200	3.7067	2.25E-04	0.0180	0.0167	1.9579	13.7398	2.25E-04	5.73E + 03	85.3358
All_Cancer	174.3105	77.5060	2.2490	0.0248	0.0067	0.0054	32.8455	5.0580	0.0248	249.7603	3.1576
Brain	2.6680	1.9913	1.3398	0.1807	0.0024	0.0011	0.8439	1.7952	0.1807	35.0175	1
Kaposi	1.7093	1.8347	0.9317	0.3524	0.0034	-0.0005	0.4100	0.8680	0.3524	88.3853	1
Stomach	1.4799	2.9595	0.5000	0.6172	3.34E-04	-0.0010	1.2542	0.2500	0.6172	5.3007	1
ALL	0.1770	0.7242	0.2444	0.8070	9.65E-05	-0.0015	0.2795	0.0597	0.8070	2.9569	1
Breast	-6.4736	10.9842	-0.5894	0.5558	4.64E-04	-0.0009	4.6549	0.3473	0.5558	6.5488	NA
Lung	-59.9607	28.6406	-2.0936	0.0366	0.0058	0.0045	12.1373	4.3830	0.0366	178.7380	NA
CML	-2.3724	0.9826	-2.4145	0.0160	0.0086	0.0071	0.3872	5.8297	0.0160	527.0733	NA
Vulva.&.Vagina	-2.9464	0.9489	-3.1052	0.0020	0.0139	0.0125	0.3776	9.6424	0.0020	2.42E + 03	NA
Thyroid	-24.9391	7.4487	-3.3481	8.54E-04	0.0148	0.0134	3.1566	11.2099	8.54E-04	2.65E + 03	NA
Penis	-1.2766	0.3645	-3.5026	5.11E-04	0.0287	0.0264	0.1034	12.2680	5.11E-04	1.52E + 05	NA
Testis	-6.8451	1.3886	-4.9294	1.02E-06	0.0315	0.0302	2.1823	24.2993	1.02E-06	3.42E + 01	NA
AML	-7.1813	1.3209	-5.4368	7.35E-08	0.0380	0.0367	0.5598	29.5592	7.35E-08	2.35E + 05	NA
Melanoma	-20,410.0796	3342.8836	-6.1055	1.65E-09	0.0475	0.0462	1416.6458	37.2775	1.65E-09	9.88E + 05	NA
Cervix	-12.7840	1.7486	-7.3111	6.82E-13	0.0667	0.0654	0.7410	53.4518	6.82E-13	1.32E + 07	NA
Pancreas	-24.8661	2.8370	-8.7651	1.24E-17	0.0931	0.0919	1.2022	76.8264	1.24E-17	2.99E + 08	NA
Liver	-35.2558	3.8607	-9.1320	6.18E-19	0.1003	0.0991	1.6361	83.3928	6.18E-19	6.57E + 08	NA
Oropharynx	-28.8908	2.9706	-9.7255	3.94E-21	0.1123	0.1111	1.2589	94.5846	3.94E-21	2.35E + 09	NA
Kidney	-49.4253	4.6700	-10.5835	1.73E-24	0.1302	0.1291	1.9791	112.0111	1.73E-24	1.48E + 10	NA
Myeloma	-23.4401	2.0434	-11.4709	3.58E-28	0.1496	0.1485	0.8660	131.5814	3.58E-28	9.97E + 10	NA

Table 8 performs a similar role for cannabis exposure. Here six tumours are significantly related with P-values less than 6.0 × 10^–5^ and minimum E-Values greater than 19.0. The cancers of interest are in order thyroid, liver, breast, bladder, pancreas and AML.

**Table 8:** Summary of Cannabis Regression Lines Slopes by Cancer and E-Value

Parameters					Model					E-Values	
**Cancer**	**Estimate**	**Std.Error**	**t-Statistic**	***P*-Value**	**R.Squared**	**Adj.R.Squared**	**S.D**	**t-Statistic**	***P*-Value**	**E-Value—Point**	**E-Value—Lower**
Thyroid	38.1264	3.6137	10.5504	2.35E-24	0.1295	0.1284	2.9671	111.3120	2.35E-24	2.40E + 05	2.74E + 04
Liver	18.7374	1.9860	9.4349	4.82E-20	0.1064	0.1052	1.6306	89.0176	4.82E-20	6.96E + 04	7.96E + 03
Breast	38.4836	5.4934	7.0054	5.50E-12	0.0616	0.0603	4.5104	49.0761	5.50E-12	4709.85	538.42
Bladder	25.5306	4.0013	6.3805	3.09E-10	0.0516	0.0503	3.2853	40.7110	3.09E-10	2355.91	269.10
Pancreas	6.9457	1.5165	4.5800	5.44E-06	0.0273	0.0260	1.2451	20.9766	5.44E-06	319.83	36.14
AML	2.7773	0.6876	4.0389	5.92E-05	0.0213	0.0200	0.5646	16.3128	5.92E-05	175.35	19.61
Brain	1.5270	1.0275	1.4861	0.1377	0.0029	0.0016	0.8436	2.2084	0.1377	9.86	1.00
Oropharynx	1.4699	1.6264	0.9038	0.3664	0.0011	-0.0002	1.3354	0.8168	0.3664	4.89	1.00
Testis	1.0020	1.1338	0.8837	0.3771	0.0010	-0.0003	2.2163	0.7810	0.3771	2.39	1.00
Hodgkins	0.4549	0.5971	0.7619	0.4463	7.76E-04	-0.0006	0.4902	0.5805	0.4463	4.08	1.00
NH_Lymphoma	1.2182	2.4060	0.5063	0.6128	3.43E-04	-0.0010	1.9754	0.2564	0.6128	2.90	1.00
Vulva.&.Vagina	0.2352	0.5173	0.4547	0.6495	3.03E-04	-0.0012	0.3802	0.2067	0.6495	2.91	1.00
ALL	0.1495	0.3740	0.3999	0.6894	2.58E-04	-0.0014	0.2795	0.1599	0.6894	2.64	1.00
CML	0.1951	0.5417	0.3602	0.7188	1.92E-04	-0.0013	0.3889	0.1297	0.7188	2.53	1.00
Melanoma	181.2616	1767.8631	0.1025	0.9184	1.41E-05	-0.0013	1451.5066	0.0105	0.9184	1.49	1.00
Stomach	-0.0371	1.5278	-0.0243	0.9806	7.90E-07	-0.0013	1.2544	0.0006	0.9806	1.19	NA
Esophagus	-0.4605	1.0392	-0.4432	0.6578	0.0003	-0.0011	0.8468	0.1964	0.6578	2.67	NA
Penis	-0.3807	0.2140	-1.7787	0.0760	0.0076	0.0052	0.1045	3.1639	0.0760	54.60	NA
CLL	-2.7590	1.4452	-1.9091	0.0566	0.0048	0.0035	1.1866	3.6447	0.0566	16.08	NA
Kidney	-5.9182	2.5755	-2.2979	0.0218	0.0070	0.0057	2.1146	5.2802	0.0218	25.02	NA
Myeloma	-3.5041	1.1365	-3.0832	0.0021	0.0125	0.0112	0.9331	9.5060	0.0021	60.46	NA
Kaposi	-2.9589	0.8886	-3.3299	0.0010	0.0415	0.0378	0.4020	11.0882	0.0010	1620.09	NA
All_Cancer	-183.9185	39.5718	-4.6477	3.97E-06	0.0281	0.0268	32.4905	21.6012	3.97E-06	344.78	NA
Lung	-72.8339	14.5848	-4.9938	7.37E-07	0.0323	0.0310	11.9748	24.9384	7.37E-07	506.24	NA
Ovary	-6.6289	1.0343	-6.4088	2.60E-10	0.0521	0.0508	0.8493	41.0721	2.60E-10	2430.83	NA
Cervix	-9.6682	0.8647	-11.1805	6.06E-27	0.1432	0.1420	0.7100	125.0043	6.06E-27	4.82E + 05	NA
Prostate	-179.6316	13.4368	-13.3687	1.05E-36	0.1929	0.1918	11.0323	178.7209	1.05E-36	5.44E + 06	NA
Colorectal	-107.1873	6.9544	-15.4129	9.43E-47	0.2410	0.2400	5.7099	237.5561	9.43E-47	5.25E + 07	NA

Table 9 performs a similar function for THC exposure. Positive findings in this table occur for nine tumours which are in order thyroid, liver, pancreas, AML, breast, oropharynx, chronic myeloid leukaemia (CML), testis and kidney. Eight cancers have minimum E-Values > 1.30. If one performs this exercise with the logarithm of THC exposure myeloma, melanoma and ALL also become significant.

**Table 9:** Summary of Δ9-TetrahydrocannabinolRegression Lines Slopes by Cancer and E-Value

Parameters						Model				E-Values	
**No**	**Cancer**	**Estimate**	**Std.Error**	**t-Statistic**	***P*-Value**	**Adj.R.Squared**	**S.D**	**t-Statistic**	***P*-Value**	**E-Value—Point**	**E-Value—Lower**
1	Thyroid	2.8220	0.2096	13.4665	3.62E-37	0.1941	2.8531	181.3454	3.62E-37	4.35	3.74
2	Liver	1.5118	0.1140	13.2611	3.36E-36	0.1893	1.5521	175.8566	3.36E-36	4.29	3.68
3	Pancreas	0.6960	0.0892	7.8048	2.01E-14	0.0741	1.2140	60.9153	2.01E-14	2.76	2.32
4	AML	0.3032	0.0404	7.5007	1.80E-13	0.0687	0.5504	56.2607	1.80E-13	2.69	2.25
5	Breast	1.9686	0.3343	5.8882	5.89E-09	0.0430	4.5517	34.6715	5.89E-09	2.33	1.93
6	Oropharynx	0.4108	0.0970	4.2358	2.56E-05	0.0221	1.3204	17.9420	2.56E-05	1.99	1.60
7	CML	0.0974	0.0317	3.0756	0.0022	0.0124	0.3862	9.4592	0.0022	1.83	1.39
8	Testis	0.3272	0.0880	3.7205	0.0002	0.0169	2.1972	13.8421	0.0002	1.55	1.33
9	Kidney	0.3065	0.1555	1.9716	0.0490	0.0038	2.1166	3.8873	0.0490	1.54	1.03
10	Bladder	0.4404	0.2473	1.7810	0.0753	0.0029	3.3664	3.1721	0.0753	1.50	1.00
11	Melanoma	169.8603	106.4342	1.5959	0.1109	0.0021	1449.0519	2.5470	0.1109	1.47	1.00
12	ALL	0.0330	0.0225	1.4708	0.1418	0.0019	0.2790	2.1633	0.1418	1.47	1.00
13	Myeloma	0.0836	0.0689	1.2137	0.2252	0.0006	0.9381	1.4731	0.2252	1.39	1.00
14	Vulva.&.Vagina	0.0347	0.0306	1.1330	0.2576	0.0004	0.3799	1.2837	0.2576	1.39	1.00
15	Penis	-0.0053	0.0117	-0.4543	0.6498	-0.0019	0.1049	0.2064	0.6498	1.27	-
16	Brain	-0.0699	0.0620	-1.1266	0.2603	0.0004	0.8442	1.2692	0.2603	1.37	-
17	Stomach	-0.1125	0.0920	-1.2226	0.2219	0.0007	1.2532	1.4948	0.2219	1.39	-
18	NH_Lymphoma	-0.3192	0.1447	-2.2064	0.0277	0.0051	1.9694	4.8682	0.0277	1.59	-
19	Hodgkins	-0.0899	0.0359	-2.5071	0.0124	0.0070	0.4884	6.2856	0.0124	1.65	-
20	CLL	-0.2244	0.0870	-2.5802	0.0101	0.0075	1.1842	6.6573	0.0101	1.66	-
21	Esophagus	-0.1719	0.0624	-2.7546	0.0060	0.0091	0.8424	7.5879	0.0060	1.70	-
22	Kaposi	-0.1915	0.0504	-3.7973	1.83E-04	0.0496	0.3996	14.4195	1.83E-04	2.47	-
23	Lung	-6.9703	0.8570	-8.1332	1.74E-15	0.0800	11.6678	66.1496	1.74E-15	2.84	-
24	All_Cancer	-21.2867	2.2921	-9.2868	1.69E-19	0.1022	31.2063	86.2456	1.69E-19	3.13	-
25	Cervix	-0.5342	0.0528	-10.1084	1.33E-22	0.1190	0.7195	102.1797	1.33E-22	3.34	-
26	Ovary	-0.7375	0.0581	-12.6901	1.47E-33	0.1761	0.7912	161.0393	1.47E-33	4.10	-
27	Colorectal	-8.5664	0.3656	-23.4321	1.75E-91	0.4225	4.9773	549.0642	1.75E-91	9.05	-
28	Prostate	-16.2170	0.6797	-23.8606	5.25E-94	0.4314	9.2532	569.3274	5.25E-94	9.33	-

Table 10 performs a similar function for cannabidiol. Here twelve cancers are implicated including in order prostate, bladder, ovary, All Cancers, colorectal, Hodgkin’s, brain, lung, Non-Hodgkin’s lymphoma, esophagus, breast and stomach cancers. In this series of tumours the nadir minimum E-Value is 30.11.

**Table 10:** Summary of Cannabidiol Regression Lines Slopes by Cancer and E-Value

Parameters						Model				E-Values	
**No**	**Cancer**	**Estimate**	**Std.Error**	**t-Statistic**	***P*-Value**	**Adj.R.Squared**	**S.D**	**t-Statistic**	***P*-Value**	**E-Value—Point**	**E-Value—Lower**
1	Prostate	575.7886	40.1598	14.3374	2.28E-41	0.2145	10.8759	205.5620	2.28E-41	1.67E + 21	2.34E + 18
2	Bladder	138.8861	11.3748	12.2100	2.15E-31	0.1651	3.0805	149.0832	2.15E-31	1.32E + 18	1.84E + 15
3	Ovary	35.7024	2.9444	12.1254	5.09E-31	0.1632	0.7974	147.0245	5.09E-31	9.91E + 17	1.38E + 15
4	All_Cancer	1081.1070	115.0938	9.3933	6.87E-20	0.1043	31.1692	88.2334	6.87E-20	1.02E + 14	1.43E + 11
5	Colorectal	213.8584	22.9037	9.3373	1.10E-19	0.1032	6.2027	87.1847	1.10E-19	8.46E + 13	1.18E + 11
6	Hodgkins	13.1216	1.7461	7.5146	1.63E-13	0.0690	0.4729	56.4697	1.63E-13	1.85E + 11	2.59E + 08
7	Brain	19.4078	3.0380	6.3884	2.94E-10	0.0505	0.8227	40.8119	2.94E-10	4.21E + 09	5.88E + 06
8	Lung	258.5289	43.9435	5.8832	6.07E-09	0.0429	11.9006	34.6121	6.07E-09	7.70E + 08	1.08E + 06
9	NH_Lymphoma	41.7851	7.1339	5.8573	7.05E-09	0.0426	1.9320	34.3079	7.05E-09	7.06E + 08	9.87E + 05
10	Esophagus	16.7282	3.1067	5.3846	9.84E-08	0.0375	0.8303	28.9943	9.84E-08	1.84E + 08	2.35E + 05
11	Breast	58.3586	17.0595	3.4209	6.58E-04	0.0141	4.6200	11.7025	6.58E-04	1.96E + 05	274.11
**12**	Stomach	12.7562	4.6084	2.7680	0.0058	0.0088	1.2480	7.6621	0.0058	2.19E + 04	30.11
13	Kaposi	4.2681	2.8705	1.4869	0.1383	0.0047	0.4089	2.2108	0.1383	2.67E + 04	1.00
14	CLL	1.4964	4.3918	0.3407	0.7334	-0.0012	1.1894	0.1161	0.7334	5.74	1.00
15	Vulva.&.Vagina	-2.3188	1.5234	-1.5221	0.1285	0.0019	0.3796	2.3167	0.1285	518.20	-
16	ALL	-3.0153	1.1413	-2.6421	0.0084	0.0096	0.2779	6.9804	0.0084	3.88E + 04	-
17	Penis	-2.0356	0.6223	-3.2712	0.0012	0.0228	0.1035	10.7008	0.0012	1.18E + 08	-
18	Melanoma	-18,124.3909	5318.6705	-3.4077	6.90E-04	0.0140	1440.3792	11.6124	6.90E-04	1.88E + 05	-
19	Cervix	-9.7353	2.8098	-3.4647	5.61E-04	0.0145	0.7609	12.0042	5.61E-04	2.28E + 05	-
20	Testis	-9.7963	2.5811	-3.7953	1.59E-04	0.0176	2.1964	14.4047	1.59E-04	1.15E + 02	-
21	Thyroid	-64.3924	11.5045	-5.5972	3.06E-08	0.0389	3.1156	31.3282	3.06E-08	2.95E + 08	-
22	Pancreas	-28.8032	4.5412	-6.3426	3.91E-10	0.0498	1.2298	40.2284	3.91E-10	3.61E + 09	-
23	Liver	-39.6678	6.2019	-6.3961	2.81E-10	0.0506	1.6796	40.9102	2.81E-10	4.32E + 09	-
24	CML	-11.8318	1.5282	-7.7425	3.58E-14	0.0803	0.3727	59.9462	3.58E-14	7.04E + 12	-
25	Oropharynx	-38.8844	4.7244	-8.2306	8.27E-16	0.0818	1.2794	67.7422	8.27E-16	2.05E + 12	-
26	AML	-17.5362	2.0075	-8.7355	1.58E-17	0.0914	0.5437	76.3096	1.58E-17	1.12E + 13	-
27	Kidney	-81.1174	7.2529	-11.1842	5.85E-27	0.1421	1.9642	125.0865	5.85E-27	4.19E + 16	-
28	Myeloma	-38.8894	3.1625	-12.2969	8.78E-32	0.1671	0.8565	151.2143	8.78E-32	1.76E + 18	-

**Fig. 8:**
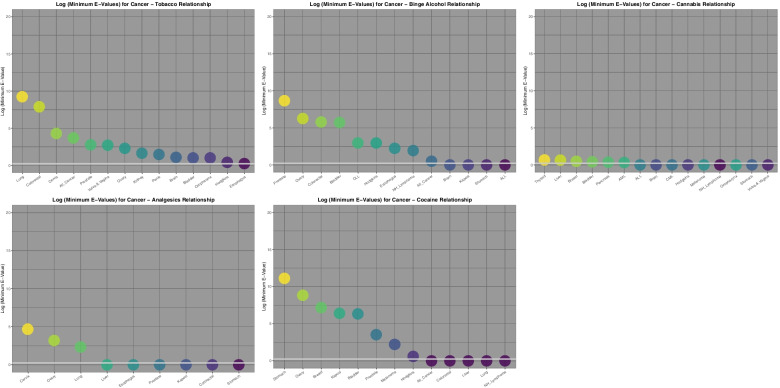
Comparative Minimum E-values regression models tumour incidence against various substances

To facilitate conceptual comparison of this mass of data Fig. 8 presents graphically the minimum applicable E-Values for these cancers by substance exposure for those cancers where a finite minimum E-Value is reported. Tumours are ordered by descending E-Value. One notes the log scale on the ordinate axis which ranges up to 10^20^. The scale is held constant across all substances to facilitate direct comparison between substances both in this graph and on the following graph. The largest minimum E-Values for tobacco, AUD, cannabis, analgesics, and cocaine are 1.76 × 10^9^, 4.67 × 10^8^, 2.74 × 10^4^, 4.76 × 10^4^ and 1.29 × 10^11^ respectively (see also Supplementary Tables 3 and 4, Excel Sheetnames “ST3 Analgesic Slopes” and “ST4 Cocaine Slopes”).

**Fig. 9:**
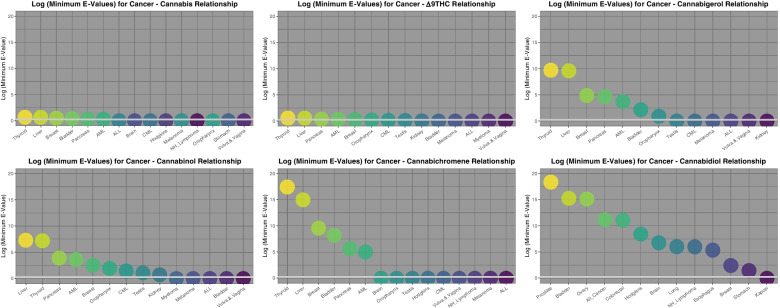
Comparative Minimum E-values regression models tumour incidence against estimates of various cannabinoid exposures

Fig 9 presents similar data for the minimum E-Values by cannabinoid exposure. The scale is held constant for consistency with the preceding graph using a log scale with a maximum of 10^20^. The most striking feature of this graph is that the minimum E-Values for cannabigerol, cannabichromene and cannabidiol dominate the graph, and are also much higher than those shown on the preceding graph which included tobacco and AUD exposure. The most dramatic minimum E-Values of all of those considered thus far relate to cannabidiol. The largest minimum E-Values for THC, cannabigerol, cannabinol, cannabichromene and cannabidiol are 4.72, 5.05X10^9^, 1.91X10^7^, 2.74X10^17^, 2.34X10^18^ respectively (see also Supplementary Tables 5, 6 and 7; Excel Sheetnames “ST5 Cannabinol Slopes”, “ST6 CBC Slopes” and “ST7 Cannabigerol Slopes”).

**Fig. 10:**
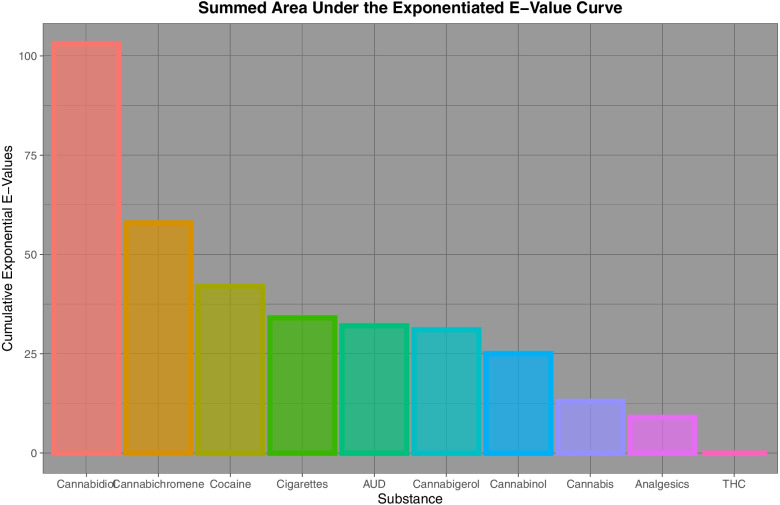
Comparative cumulative sum of the regression model minimum E-value exponents by substance

Fig. 10 summarizes these E-Value graphs by illustrating as a bar graph, the cumulative exponents of the E-Values for each substance. This is a simple way of integrating the area under the E-Value curve apparent for each substance. In reality any summary statistics could have been chosen for comparison (e.g. median, interquartile range, range etc.) but it was felt that use of the sum had the major advantage of integrating the area underneath the E-value curve and therefore most closely quantifying the key parameter of interest. From this graph it is clear that for the cancers selected, the area under the curve for cannabidiol and cannabichromene (103 and 58) are considerably larger than that for tobacco and AUD (34 and 32). Other values for the graph are shown in Table 11.

**Table 11:** Cumulative E-Values Exponents of Regression Lines by Substance

**Substance**	**Summed Exponents of E-Values**
Cannabidiol	103
Cannabichromene	58
Cocaine	42
Cigarettes	34
AUD	32
Cannabigerol	31
Cannabinol	25
Cannabis	13
Analgesics	9
THC	0

## Discussion

### Main results

The main result of this survey and overview is that strong continuous bivariate relationships are noted between the incidence of many cancers and cannabinoids including cannabidiol to an extent comparable to and indeed exceeding that seen with tobacco and alcohol. Whilst positive regression slopes were seen for 9 and 13 cancers for tobacco and AUD exposure, the applicable numbers for cannabis, THC, cannabidiol, cannabigerol, cannabinol and cannabichromene exposure were 15, 14, 13, 13, 15 and 15 cancers respectively (Tables 3 and 4). Elevated minimum E-Values occurred for tobacco and alcohol exposure for 14 and 9 cancers and for 6, 9, 12, 6, 9 and 7 cancers in association with cannabis, THC, cannabidiol, cannabichromene, cannabinol and cannabigerol exposure (Tables 6, 7, 8, 9 and 10 and Supplementary Tables 3, 4, 5, 6 and 7). Compared to tobacco and AUD exposure which have largest minimum E-Values of 1.76 × 10^9^ and 4.67 × 10^8^, the largest minimum E-Values for exposure to THC, cannabigerol, cannabinol, cannabichromene and cannabidiol were 4.72, 5.05 × 10^9^, 1.90 × 10^7^, 2.74 × 10^17^, 2.34 × 10^18^ respectively (Fig. 9 and 10 and above cited Tables). The summed exponents of the minimum E-Values for cannabidiol and cannabichromene were 103 and 58 compared to 34 and 32 for tobacco and AUD. The summed exponents for cannabigerol, cannabinol, cannabis and THC were 31, 25, 13 and 0 respectively. These results are in close concordance with the results reported in an accompanying report for the categorical analysis [69].

Causality in the bivariate results was implied by the high E-Values documented.

Hence the present findings argue strongly for the significance of cannabis and cannabinoids as serious bona fidé carcinogens in the US environment. These findings are strengthened by results in accompanying reports [69, 70] showing that the reported bivariate changes are robust to adjustment, fulfil quantitative epidemiological criteria for causality, and for prostate and ovarian cancer demonstrate a supra-linear sigmoidal dose–response relationship with carcinogenic outcomes so that rising doses of cannabinoid exposure generate disproportionate cancerogenic outcomes.

### Interpretation

Some of these findings are particularly noteworthy. All cancers as a group were noted to rise with both tobacco exposure and with cannabidiol exposure. It is concerning that another major carcinogen appears to have been identified, which at present is being consumed virtually without restriction in many parts of USA, Canada and elsewhere.

It is also concerning that at least judged by the area under the E-Value curves that cannabidiol and cannabichromene (cumulative minimal E-Value exponents of 103 and 58) were shown to be a more powerful environmental carcinogens than tobacco and alcohol (34 and 32).

From the findings with AML (present report and [87]) and other pediatric cannabis-related tumours [12, 18–20, 54, 88–90] real concerns exist that widespread cannabinoid exposure may lead to a multigenerational epidemic of cancer. This is supported by recent US history with the rate of all childhood cancer rising 49% and the rate of acute lymphoid leukaemia, the commonest cancer of childhood, rising 94% in the period 1975 to 2018 [48]. This view is closely concordant with a recent report describing cannabis exposure as a primary driver of USA pediatric cancers [55] and of the commonest cancer of childhood acute lymphoid leukaemia [53]. From the very clear findings with testicular cancer it would appear that the usual course of oncogenesis in some stem cell niches may be greatly accelerated [51].

Given the rising level of cannabinoid exposure in the US community, its entry into the food chain seems inevitable. Indeed in some states such as Kentucky and Mississippi this already appears to be occurring [91]. One of the pressing needs in the field therefore is for the development of reliable biomarkers possibly derived from epigenomic or glycomic metrics so that cannabinoid exposure can be quantified formally and analyzed as a continuous variable as has been previously suggested [92]. This would greatly improve epidemiology and surveillance in this field, reduce the numbers required and increase the geospatial precision with which temporal trends can be mapped and surveilled.

Higher precision geotemporospatial mapping of cancer trends is also indicated where such data is available.

### Causal assignment

E-values have been used extensively in the present report. In the literature E-Values greater than 1.25 are said to be linked with causality [81]. It is worth noting that the minimum E-Value for the association between tobacco smoke and lung cancer is 9. This places the greatly elevated E-Values highlighted in this report in a proper context. The methodology employed here has also been validated *en passant* in that many tobacco-related cancers including lung, colorectum, all cancer, vulva and vagina, penis, bladder, oropharynx and esophagus, were correctly identified as such by the methodology adopted.

The findings relating to causal analysis in the present report are further strengthened by the accompanying categorical analysis and the detailed presentation of inverse probability weighted regression models and geospatiotemporal modelling in accompanying reports [69, 70].

### Specific cancers

#### Breast

Breast cancer is the commonest cancer. It was noted to be linked with cannabis, THC, cannabidiol, cannabinol, cannabichromene, cannabinol and cannabigerol. It would seem to be a major public health concern that the commonest cancer is linked with an increasingly common environmental exposure.

#### Bladder and prostate

It is interesting that bladder and prostate cancer are linked with cannabis and cannabidiol exposure, as bladder cancer has previously been linked with tobacco smoking. In the case of tobacco the causative action is believed to be the prolonged time tobacco-derived carcinogens spend in contact with the transitional epithelium of the bladder [86]. It is known that many of the carcinogens of tobacco are also found in cannabis smoke. Accumulation of urinary CBD and THC metabolites over ten days of cannabis consumption has been documented [93]. It may be that the association of bladder cancer with cannabidiol documented above rests on a similar mechanistic basis. Presumably similar actions are in play in relation to cannabis and cannabidiol urocarcinogensis.

#### Testis

Testicular cancer has been linked with cannabis use in prior investigations by all four studies to have examined this issue [8–11]. It was seen in the present work to be linked with THC exposure but not cannabidiol exposure. The involvement of THC with testicular cancer is a cause for concern for two reasons in that it is a germinal epithelium and so genotoxic changes there could well be passed on to subsequent generations. Secondly the extensive literature on the pathogenesis of testicular cancer states quite repeatedly and emphatically that testicular cancer is thought to arise from changes which occur in utero which then become manifest due to the hormonal surge of puberty [18, 54]. Since what is being witnessed at present is that cannabis use is being reflected relatively quickly in higher rates of testicular cancer this necessarily involves a profound telescoping and contraction of the usual decades long pathogenic pathways of testicular cancer from several decades to several years. This implies that, at least in the testis, THC must be acting a powerful carcinogen indeed. One notes indeed that since 1975 the age-adjusted rate of testicular cancer across USA has doubled [48]. It may also be a hint that relatively abrupt mechanisms such as those mentioned above in relation to myeloid malignancies may also be operating in this germ cell context [94].

#### Ovary

As was shown the ovary is also implicated in cannabidiol exposure and carcinogenesis. This is also concerning. The ovary of course contains the female germinal epithelium. These findings imply that both male and female germinal epithelium are subject to cannabis induced genotoxicity and / or epigenotoxicity and carcinogenesis. The prospect of offspring who have been subject to mutagenic and potentially teratogenic actions in both parental gonads is very concerning indeed, particularly as it is well established that epigenomic changes are heritable for multiple generations [95, 96].

#### Liver

Liver cancer was one of the two cancers most affected by cannabis, THC, cannabinol cannabigerol and cannabichromene exposure. This is a provocative finding as cannabis has previously been linked with exacerbating liver inflammation and inducing cirrhosis, especially in patients with other risk factors for hepatic disease [97]. This is consistent with the frequently pro-inflammatory action of cannabinoids binding at cannabinoid type 1 (CB1R) receptors. This finding implies that THC and its related cannabinoids is linked with not only hepatic proinflammatory processes but that it is linked with persistent chemical hepatitis to the point of neoplasia.

The endogenous endocannabinoid anandamide along with its natural receptor the cannabinoid type 1 receptor (CB1R) are known to be normally involved in hepatic lipogenesis, insulin resistance and glucose intolerance and to be strongly upregulated during normal liver regeneration following partial hepatectomy or major liver injury and confer on the liver a remarkable degree of regenerative capacity [98, 99]. Anandamide (AEA) stimulates CB1R synthesis which further stimulates AEA release in a autoinductive loop typical of tumour promoting growth factors [99]. CB1R also stimulates multiple oncogenic pathways including the key master transcription factor Forkhead Box M1 (FOXM1) [99]. FOXM1 stimulates indoleamine 2,3 dioxygenase (IDO2) which stimulates T-reg cells which are immunosuppressive and induce tumour tolerance. CB1R also interacts directly with the pro-oncogenic Growth Factor Receptor Bound Protein 2 (GRB2) [100–102] and stimulates its interactome which signals activation to many oncogenic nuclear genes including RAS [99]. CB1R and IDO2 also stimulate angiogenesis and the ingrowth of new vessels to the developing tumour [99].

Whist it is noted that cannabinoids have both tumour stimulatory and tumour inhibitory actions it is also pointed out that the tumour stimulatory actions occur at nanomolar concentrations close to the dissociation constants of cannabinoids whilst the tumour suppressive actions occur at much higher micromolar concentrations [98, 99].

Cannabidiol alone was also found to induce liver hypertrophy even at the low concentration of 17 μM in a recent study [103].

Since the liver is a major metabolic organ and controls the central metabolic milieu, and since its inflammatory state is a key regulator of many metabolic pathways both in the liver and systemically in immune and other cells, this implies that hepatic inflammation is linked with a dysmetabolic state systemically throughout the organism, This dysmetabolic and systemic proinflammatory state is itself known to be linked with pro-aging processes including oncogenesis [104–108]. Moreover the oxidative action of cannabidiol and related cannabinoids on DNA bases is greatly increased in oxidizing environments such as cellular inflammation [43]. Inflammation is known to increase the activity of retrotransposons repeat pseudogenes which are endogenous to the human genome and makes the “jumping genes” jump [109, 110]. This increases genomic instability and has been linked with tumour invasiveness, growth rate and metastasis [109–111]. Some of the genomic material spills into the cytoplasm where it stimulates innate immunity directly via the cytoplasmic GMP-AMP Synthase and the Stimulators of Interferon Gamma (cGAS-STING) pathway [111–115]. These processes thus set up positive feedback loops as inflammation causes increased mutation and genomic destabilization which stimulates further inflammation [99, 116]. This positive feedback loop between inflammation and genomic instability may be a key driver of the many case series reporting a link between early and high dose cannabis use and the development of aggressive highly metastatic tumours in patients of younger ages [117–120]. Complex interplays have been demonstrated between metabolic state, immunophenotype, immune cell differentiation, epigenetic state and tumourigenesis [121].

## Non-Hodgkins lymphoma

### Histone 1 (H1)

It was shown as long ago as 1981 that THC and cannabinol inhibit H1, H2a and H2b histone synthesis by 50% after acute administration to cultured cells [122]. Importantly these investigators also showed that the acetylated forms of these histones, which in general are permissive for gene transcription were similarly reduced to 50–60%.

Whist most of the histones are core proteins at the centre of the histone octamer, H1 is a linker protein which sits like a clasp or clamp near the entry and exit of the DNA strand to hold the whole assembly together [123, 124]. H1 undergoes many post-translational modifications including phosphorylation, acetylation, methylation, citrullination, ubiquitylation, formylation, denitration, ADP-ribosylation, crotonylation, and lysin-2-hydroxyisobutyrylation, many of which have functional significance [124].

H1 also interacts powerfully with the genome repressive machinery particularly (Polycomb Repressive Complex 2) to recruit genome repression and to help form heterochromatin which is transcriptionally inactive [125]. Hence H1 is a key determinant of gene silencing [126]. Indeed its knockdown has been shown to lead to hyperactivation of B-cells in the germinal centres (GC) of lymph nodes where it acts to make genes more available for transcription, increases the activation of stem cell genes, provides fitness and self-renewal advantages to GC B-cells, and thereby launches aggressive B-cell neoplasias. Indeed H1 mutations have been identified in over 80% of B-cell lymphomas [127] and found to be mainly loss-of-function mutations. Data indicated that H1 is an absolute requirement to sequester genes in transcriptionally inactive compartments (the B-nuclear compartment). B-cells are thought to be particularly sensitive to this action as hypermutation is a normal part of their repertoire following activation as it is the mechanism by which they produce antigenic variation in their B-cell receptors.

That is to say that cannabinoid exposure partly phenocopies genetic allelic ablation of H1. Together with the other genetic, epigenetic, chromosomal and metabolic effects of cannabinoids outlined above, these effects may explain the presence of the signal for Non-Hodgkin’s lymphoma in the present epidemiological analysis.

### AML and ALL

AML has previously been linked with parental cannabis consumption [18, 54]. In the present study AML was found to be elevated with cannabis exposure and AML and CML were noted to be elevated by THC exposure. ALL is mainly a pediatric cancer and it has been linked to inherited genotoxicity [26, 27]. It was recently shown epidemiologically to be causally linked to environmental cannabis exposure [53]. The increase of the ALL incidence rate by cannabis and THC necessarily implies transgenerational teratogenesis, mutagenesis and oncogenesis. This issue was further heightened by a recent report noting that cannabis consumption is a major driver of the 50% rise in total pediatric cancer in the USA since 1970 [55]. This is a grave concern indeed as it indicates not only heritable mutagenesis but heritable carcinogenesis. The number of generations for which such inheritance can continue has not been defined at the time of writing. It is believed however that epigenetic changes can be inherited for three to four generations [95, 96] which translates to about the next one hundred years. Moreover it was recently shown that myeloid malignancies can be suddenly oncogenically transformed by relatively abrupt clonal sweeps due to specific genotoxic stressors where a minor clone collects extra mutations which suddenly sweep it into clonal dominance and drive overall tumourigenesis and malignant behaviour [94].

It was shown recently for myeloid malignancies that they tend to collect epimutations which often affect epigenomic signalling genes [94]. Clones with the most advantageous collection of mutations out-perform others and can become dominant within the tumour a phenomenon which can happen either spontaneously or as a result of treatment imposed tumour stress. Moreover this can happen relatively abruptly in what has been referred to as “clonal sweeps” across the tumour [94].

### Mechanisms

The cellular and molecular mechanisms underlying these epidemiological relationships outlines in the above analyses are outlined further in the second and third papers in the present series.

### Generalization

We feel that our results are widely generalizable for several reasons. As noted above they are internally very consistent both with each other and with much known evidence external to this study. The cancer data used are derived from census samples from all US states. The drug exposure data is taken from a well authenticated and widely studied nationally representative survey which has been operating for several decades. The bivariate analysis is at once conceptually simple yet very powerful especially when paired with E-Value calculations. For prostate and ovarian cancer bivariate results were verified by further causal regression and space–time modelling which confirmed the bivariate results and demonstrated overall robustness to multivariable adjustment. One of the major result outputs from the present study was E-Values which are a major pillar of causal inference. We are of the view that the large US dataset represents an ideal context within which to address the present concerns. In that the present results demonstrate causal relationships we are confident that they could be widely reproduced with the sole caveat that in nations where cannabis use is more widespread we would expect the findings to be more dramatic provided that the data collection systems are sufficiently accurate.

### Strengths and limitations

It is important that this study be read in parallel with the other two papers in this series [69, 70]. This study has several strengths. We used a large national cancer census dataset. Age adjusted rates derived from CDC, SEER and NCI were employed. The drug dataset was from a large well validated nationally representative dataset. The bivariate statistics were straightforward yet, when harnessing the power of E-values they were powerful to address causality directly. These studies were internally and externally consistent with known data both on tobacco-related cancer and on cannabis-related cancer. Panelled graphs were used to allow the simultaneous display of results for direct comparison across many cancers. Together with other papers in the present series [69, 70] the present report strongly indicts population level cannabinoid exposure in cancer aetiopathogenesis.

In common with most epidemiological studies this study did not have available to it individual level participant data. State-level cannabinoid exposure had to be estimated as described as state level data itself was not directly available to the present investigators. This study is an epidemiological study and thus is not able to formally prove with formal experimental rigor the causal nature of the relationships indicated from these studies at the level of population health. However these results do indicate detailed mechanistic studies in many cell lines and tumour models. Another issue of considerable interest is the possible role of synthetic cannabinoids as genotoxins. In the absence of spatiotemporal data on this issue we are unable to comment on this increasingly important matter. However several lines of evidence suggest that they are likely to be implicated. Several recent studies implicate many cannabinoids in genotoxic activities [37, 38, 43, 51–53, 55, 87, 91, 128–130]. Long ago the genotoxic action was found to reside in the polycyclic olevitol nucleus of the cannabinoids with little modulation by the various side chains [36]. And several other studies implicate synthetic cannabinoids in genotoxicity [131–137]. Overall therefore we feel that this is a fertile and important area for further laboratory based investigation and epidemiological surveillance.

Furthermore this was also an ecological study. It is therefore potentially susceptible to the short-comings typical of ecological studies including the ecological fallacy and selection and information biases. Within the present paper we began to address these issues with the use of E-values in all Tables. This issue is further addressed by the detailed pathophysiological mechanisms which have been described above, by mention of other countries where many of the same findings have been made, and with the use of inverse probability weighting in multiple regression models and further extensive application of E-values in Parts 2 and 3 of the present series of papers.

## Conclusion

In conclusion this overview of 28 selected cancers showed strong bivariate evidence that cannabis and several cannabinoids were associated with multiple cancers. All cancer incidence was associated with cannabidiol exposure. Breast cancer, the commonest cancer, was associated with tobacco, cannabis, THC, cannabidiol, cannabinol, cannabichromene and cannabigerol exposure. The pediatric cancer AML was linked with THC exposure. It is also presumptive evidence of transgenerational transmission of oncogenesis. Testicular cancer, previously linked with cannabis exposure, was found to be linked with THC exposure. THC greatly accelerates the course of testicular carcinogenesis by several decades. The area under the cumulative exponential E-Value curve for tobacco, AUD, cannabis, THC, cannabidiol, cannabichromene, cannabinol and cannabigerol was 34, 32, 13, 0, 103, 58, 25, 31 indicating that cannabidiol appears to be most strongly implicated in environmental carcinogenesis of the substances studied. The clear implication from this work and its accompanying reports [69, 70] including the suggested extensions is that community penetration of cannabinoids should be carefully restricted not only as a matter of public health and safety including importantly integrity of the food chain, but also as a non-negotiable investment in the genomic health and onco-protection of multiple coming generations in a manner precisely analogous to that of all other seriously genotoxic agents. Particular concerns relate to the movement of increasing sections of the community into higher dose ranges of cumulative cannabinoid exposure in the context of exponentiation of genotoxic dose-responses which has now been convincingly demonstrated both in the laboratory and in epidemiological studies of human populations.

## Supplementary Information

**Additional file 1:** **Supplementary Table 1.** Substance Slopes for all Cancers. **Supplementary Table 2.** Cannabinoid Slopes for all Cancers. **Supplementary Table 3.** Slopes of Cancers by Analgesic Exposure. **Supplementary Table 4.** Slopes of Cancers by Cocaine Exposure. **Supplementary Table 5.** Slopes of Cancers by Cannabinol Exposure. **Supplementary Table 6.** Slopes of Cancers by Cannabichromene Exposure. **Supplementary Table 7.** Slopes of Cancers by Cannabigerol Exposure.

## Data Availability

All data generated or analysed during this study are included in this published article and its supplementary information files. Data has been made publicly available on the Mendeley Database Repository and can be accessed from this URL http://dx.doi.org/10.17632/dt4jbz7vk4.1.

## Abbreviations

AEA: Anandamide
AIAN: American Indian / Alaska Native
ALL: Acute Lymphoid Leukaemia
AML: Acute Myeloid Leukaemia
AUD: Alcohol Use Disorder
CB1R: Cannabinoid Type 1 Receptor
CBC: Cannabichromene
CBD: Cannabidiol
CBG: Cannabigerol
CBN: Cannabinol
CDC: Centers for Disease Control, Atlanta, Georgia
cGAS-STING: Cytoplasmic GMP-AMP Synthase and the Stimulators of Interferon Gamma
CLL: Chronic Lymphoid Leukaemia
CML: Chronic Myeloid Leukaemia
DEA: Drug Enforcement Agency
E-Value: Expected Value
FOXM1: Forkhead Box M1
GC: Germinal Centre
IDO2: Indoleamine 2,3 Dioxygenase
mEV: Minimum E-Value (2.5% Threshold Level)
NCI: National Cancer Institute
NHL: Non-Hodgkins Lymphoma
NHPI: Native Hawaiian / Pacific Islander
NPCR: National Program of Cancer Registries
NSDUH: National Survey of Drug Use and Health
SAMHDA: Substance Abuse and Mental Health Services Administration
SAMHSA: Surveillance Epidemiology and End Results Program
SEER: Surveillance epidemiology and End Results Program
THC: Δ9-Tetrahydrocannabinol
